# 

**Published:** 1908-06-27

**Authors:** 


### CONTENTS.

| | PAGE |
|---|---|
| **Prevention or Cure** | 327 |
| **The Research Defence Society** | 328 |
| **Annotations—** | |
| What is Margarine?—The Sleeping Canopy—The Motor 'Bus Nuisance | 329 |
| **Medical Opinion and Movement** | 330 |
| **Hospital Clinics—** | |
| Conjunctivitis in Children. By J. Herbert Parsons, D.Sc., F.R.C.S. | 333 |
| **Special Article—** | |
| Medical Defence Societies | 335 |
| **Medicine—** | |
| Acute Rheumatism and Pneumonia | 337 |
| The Sequelæ of Enteric Fever—IV. | 338 |
| **Surgery—** | |
| Some Aspects of Intestinal Obstruction | 339 |
| **Gynæcology—** | |
| Urethral Caruncle and Urethral Prolapse | 340 |
| **Public Health and Hygiene—** | |
| Clean Milk, Pasteurised Milk, and Sterilised Milk | 341 |
| **Mental Diseases—** | |
| Acute Mania | 342 |
| **The Royal Army Medical Corps Section—** | |
| A Proposed Nursing Service for the Territorial Army | 343 |
| **Dermatology—** | |
| Tuberculides | 344 |
| **The Practitioner's Relaxations and Hobbies—** | |
| Print-Collecting as a Hobby | 345 |
| **Practical Notes on Diagnosis and Treatment** | 346 |
| **Hospital Administration—** | |
| Graduate Study on the Continent—Germany | 347 |
| Nauheim Treatment in London | 348 |
| New Appliances and Things Medical | 348 |
| News and Coming Events | 349 |
| **Nursing Administration—** | |
| The Workroom | 351 |
| **The Graduates' Medical Schools** | 352 |
| **Editor's Letter-Box—** | |
| Visit of French Doctors | 352 |

